# *Anisakis pegreffii* Larvae in *Sphyraena viridensis* and Description of Granulomatous Lesions

**DOI:** 10.3390/ani11123449

**Published:** 2021-12-03

**Authors:** Giovanni De Benedetto, Alessia Giannetto, Kristian Riolo, Carmelo Iaria, Emanuele Brianti, Gabriella Gaglio

**Affiliations:** 1 Department of Veterinary Sciences, University of Messina, 98168 Messina, Italy; ebrianti@unime.it (E.B.); ggaglio@unime.it (G.G.); 2 Department of Chemical, Biological, Pharmaceutical and Environmental Sciences, University of Messina, 98166 Messina, Italy; agiannetto@unime.it (A.G.); kristian.riolo@unime.it (K.R.); ciaria@unime.it (C.I.)

**Keywords:** *Anisakis pegreffii*, Anisakidae, *Sphyraena viridensis*, inflammatory reaction, gastric granuloma, humans

## Abstract

**Simple Summary:**

Fish-borne zoonoses are caused by bacteria and parasites, while no viral fish-borne zoonoses have been reported, to date. Regarding zoonoses caused by parasites, *Anisakiasis* is one of the most important, with *Anisakis simplex* and *Anisakis pegreffii* agents in the central Mediterranean Sea. Humans can be infected by accidental ingestion of third-stage larvae in raw, undercooked or improperly processed fish or cephalopods. After ingestion, the larvae migrate from the gastrointestinal tract to gastrointestinal tissue, causing pain and, subsequently, inflammatory reaction leading to eosinophilic granuloma. This kind of reaction has not been described to date, in fish, and the aim of this study is to describe gastric wall lesions caused by *A. pegreffii* in *Sphyraena viridensis* and to compare them to those reported in humans, which appear macroscopically identical, albeit showing significant microscopic differences.

**Abstract:**

The aim of the present study was to describe gastric granuloma caused by *Anisakis pegreffii* in *Sphyraena viridensis* caught in the central Mediterranean Sea. Sixty-eight *S. viridensis* specimens were collected from different fish markets on the east coast of Sicily. Coelomic organs were observed both macroscopically and with the aid of stereomicroscope. Parasite specimens and lesioned tissues were collected for identification, histological and molecular analyses. Twelve specimens (*p* = 17.6%) were positive for the presence of nematode larvae, morphologically identified as larvae of *Anisakis* sp., with values of mean abundance and mean intensity of 0.9 and 4.8, respectively. One large female specimen showed massive parasite infection associated with nodular lesions of the gastric wall. By histology, several nematode larvae encysted through the gastric wall were found. The parasite bodies were surrounded by a granulomatous reaction made up of macrophages, epithelioid cells, some lymphocytes and an external connective sheet. Molecular analysis of *18S rRNA* and *cox2* genes from *Anisakis* sp. collected larvae, identified them as *A. pegreffii*. The lesions here described, though macroscopically superimposable on human eosinophilic granuloma, microscopically showed significant differences in the inflammatory cells involved and in the type of immune reaction.

## 1. Introduction

Four species of Sphyraenidae have been reported in the Mediterranean Sea: *Sphyraena chrysotaenia* and *Sphyraena flavicauda* along the Red Sea coast, and *Sphyraena viridensis* and *Sphyraena sphyraena* in the central Mediterranean Sea [1,2].

Yellowmouth barracuda (*S. viridensis*, Cuvier, 1829) is a common coastal pelagic predator in the central Mediterranean Sea [3], where it can reach 90 cm in length, but generally ranges from 25 to 50 cm in length. It is a gregarious species, even if solitary specimens have been described [1]. According to the state of growth, *S. viridensis* feeds mainly on crustaceans and fish [1]. In the Mediterranean Sea it has been described from 50 m depth to the water surface where *S. viridensis* moves to feed [1]. The mating season extends from September to April and the first stages of development are exclusively planktonic [1]. Though several studies have been carried out on the occurrence of this fish species in different areas, e.g., Azores Archipelago, Madeira and Cape Verde [4,5,6,7,8], limited and dated information on its biology and distribution in the central Mediterranean Sea are available [3].

*Sphyrena viridensis*, as a result of its diet based on benthonic fish and cephalopods, might be exposed to infection by *Anisakis* spp. [9]. Nematodes belonging to the Anisakidae are characterized by an heteroxenous life cycle, which involves various organisms of the marine ecosystem. Small crustaceans, belonging to the order Euphausiacea, represent the first intermediate hosts, fish and cephalopods are intermediate/paratenic hosts, where *Anisakis* larvae develop into the infesting third stage (L3). Adult parasites have been found in the stomach of marine mammals, such as cetaceans, that prey on intermediate hosts [10]. In fish, larvae are localized on the serosa of the coelomic organs, and in some species, also in the muscle [11].

The species *Anisakis simplex* and *Anisakis pegreffii* have been identified as the causative agents of human anisakidosis [12,13]. Humans act as accidental intermediate hosts, and become infected when eating raw or undercooked fish or cephalopods carrying L3 larvae [14]. In humans, L3 larvae move from the gastrointestinal tract to the gastrointestinal mucosa up to the submucosa, initially causing pain and subsequently a granulomatous reaction identified as eosinophilic granuloma [11]. The immune response during helminth infection is supported by T helper 2 (Th2) lymphocytes, with release of cytokines, such as interleukin 3 (IL3), IL4, IL5, IL9 and IL13, eosinophilic granulocytes, mast cells and activated macrophages [15]. Since human is an accidental host, it is possible that the immune response during nematode infection is not properly modulated in this kind of atypical host [16]. Indeed, allergic-like reactions associated with *Anisakis* infections have been reported over the years [17,18]. The first case of human infestation was reported in the Netherlands in 1960 [19]. About 20,000 cases referable to this zoonosis have been reported in humans worldwide, with 90% of them in Japan [14]. In Italy, several cases of anisakiasis caused by *A. pegreffii* have been reported along the meridional coasts, where there is an increased consumption of raw or undercooked fish [20,21].

Granulomatous reactions have been reported, but not deeply described in European sea bass (*Dicentrarchus labrax*) and gilthead sea bream (*Sparus aurata*) after experimental infection by *Anisakis pegreffii* larvae [22,23] and in wild fish such as John dory (*Zeus faber*) sampled from the central Mediterranean Sea [24].

It is noteworthy that *A. pegreffii* has been found as the main nematode species infecting *S. viridensis* [9] in the western Mediterranean Sea; however, the typical lesions observed in human hosts including gastric eosinophilic granuloma have not been compared with granuloma caused in *S. viridensis* to date.

In this study, we describe gastric lesions and granuloma caused by *A. pegreffii* in *S. viridensis* and compare them with those reported in humans. This study represents the first report of a human-like gastric lesion caused by *A. pegreffii* infection in fish, and it provides clues for a better understanding of the pathogenic pathway power of this nematode infection in intermediate fish hosts.

## 2. Materials and Methods

### 2.1. Fish Sampling

During this survey, 68 *S. viridensis* specimens, caught in the Mediterranean Basin (FAO area 37.2.2), were collected from different fish markets in the east coast of Sicily. All collected specimens were immediately stored at +4 °C and transferred to the laboratory of Parasitology and Parasitic Diseases, University of Messina to perform necropsy and parasitological analysis. All fish were measured (total length, TL) and weighed (body weight, BW) (PBA220, Mettler Toledo, Columbus, OH, USA, accuracy of 1 g), and measurements were recorded.

### 2.2. Parasitological Analysis

The fish were dissected and examined for *Anisakis* spp. larvae presence according to Arthur & Albert [20]. Briefly, coelomic organs were observed both macroscopically and with a stereomicroscope (Stereo Discovery.V12 Zeiss, Jena, Germany), as described by Piras et al. [9]. Parasites found were washed in physiological solution and fixed in 70° ethanol. Samples used for molecular analysis were stored at −80 °C. Morphological larvae identification was performed using morphological keys suggested by Berland B. [25] and Sonko et al. [26], with the aid of an optical microscope (Axioskop 2 plus Zeiss) after glycerin diaphanization. Epidemiological indices of prevalence (*p*, %), mean abundance (MA) and mean intensity (MI) were calculated according to Bush et al. [27]. Pearson’s correlation was calculated to evaluate the relationship between *S. viridensis* body weight (log transformed) and *A. pegreffii* load (number of larvae per subject). Statistical significance was set at *p* values < 0.05. Statistical analyses were executed using the software GraphPad Prism version 9.0 (GraphPad Software, San Diego, CA, USA).

### 2.3. Histological Analysis

For histological evaluations, macroscopically visible lesions were excised and fixed in buffered 10% formalin solution for 48 h, routinely processed and paraffin-embedded at 56 °C. Four-micron thick sections were cut and routinely stained with hematoxylin and eosin (H&E) [28].

### 2.4. Molecular Analysis

Total DNA was extracted from parasite specimens using the Nucleo Spin Plant Ⅱ kit (Macherey-Nagel, Düren, Germany) according to the manufacturer’s instructions. DNA quantity, purity and integrity were verified by UV absorbance measurements at 260 and 280 nm (NanoDrop 2000, Thermo Scientific, Wilmington, MA, USA). Polymerase chain reactions (PCR) were carried out for the identification to species level using two specific primer sets (Table 1) and Taq DNA Polymerase Recombinant kit (Invitrogen).

The *18S rRNA* gene from *Anisakis* spp. was amplified using the primers Nem_18S_F and Nem_18S_R from Floyd et al. [29] and the following PCR conditions: after a first step of 94 °C for 5 min, DNA was subjected to 35 cycles of 94 °C for 30 s, 54 °C for 30 s and 72 °C for 1 min, with a final extension of 72 °C for 10 min. The mitochondrial *cytochrome C oxidase subunit II* (*cox2*) gene was amplified using the primers 211 and 210 from Nadler and Hudspeth [30]. The PCR conditions were as follows: 94 °C for 3 min, 35 cycles of 94 °C for 30 s, 46 °C for 1 min and 72 °C for 90 s, followed by post amplification at 72 °C for 10 min [31]. The PCR products were resolved by 2.0% agarose gel electrophoresis and amplicons of the expected size were purified using the E.Z.N.A Gel Extraction Kit (OMEGA), according to the manufacturer’s protocol. DNA sequencing of the purified fragments was performed on the Applied Biosystems 3730 DNA Analyzer (Thermo Fisher Scientific, Waltham, MA, USA), using both forward and reverse primers for each gene analyzed.

The sequences of *18S rRNA* (926 bp) and mtDNA *cox2* (629 bp) obtained from the *Anisakis* spp. specimens from *S. viridensis* were analyzed by BLASTN similarity search against the National Center for Biotechnology Information (NCBI) database (https://blast.ncbi.nlm.nih.gov/Blast.cgi, accessed on 4 June 2021). The *cox2* sequences were aligned with previously characterized sequences of other known *Anisakis* species and deposited in GenBank [32], by ClustalW, carried out using MEGA X software [33]. Phylogenetic analyses on the *cox2* sequence data sets were carried out by MEGA X using Maximum Parsimony (MP) and Neighbor-Joining (NJ), based on p-distance. Reliabilities of phylogenetic relationships were evaluated using nonparametric bootstrap analysis with 1000 replicates for MP and NJ trees. Bootstrap values exceeding 70 were considered well supported [34].

## 3. Results

**Figure 1:**
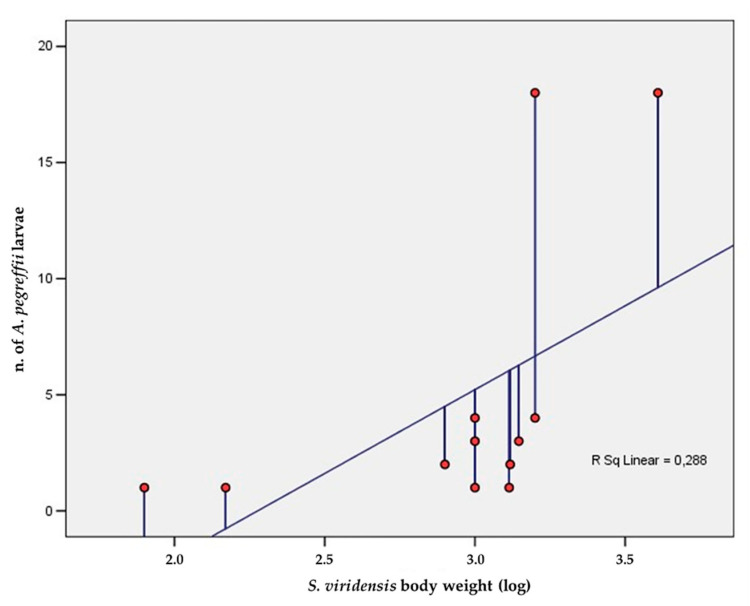
Correlation between *Anisakis pegreffii* larvae load and logarithm of *S. viridensis* body weight.

Host specimens sampled (48 males and 20 females) had a mean length of 68 ± 12.8 cm and a mean weight of 1110 ± 638.7 g. Twelve specimens (*p* = 17.6%) scored positive for the presence of nematode larvae with values of MA and MI of 0.9 and 4.8, respectively. The total count of larvae ranged from 1 to 18 per specimen Morphologically, the nematode larvae were identified as *Anisakis* Type I larvae. No significative differences about parasite load were found between male and female specimens; Pearson’s correlation test showed a positive correlation (R = 0.5 *p* < 0.05) between the *Anisakis* larvae load and *S. viridensis* body weight considered as logarithm (Figure 1).

**Figure 2:**
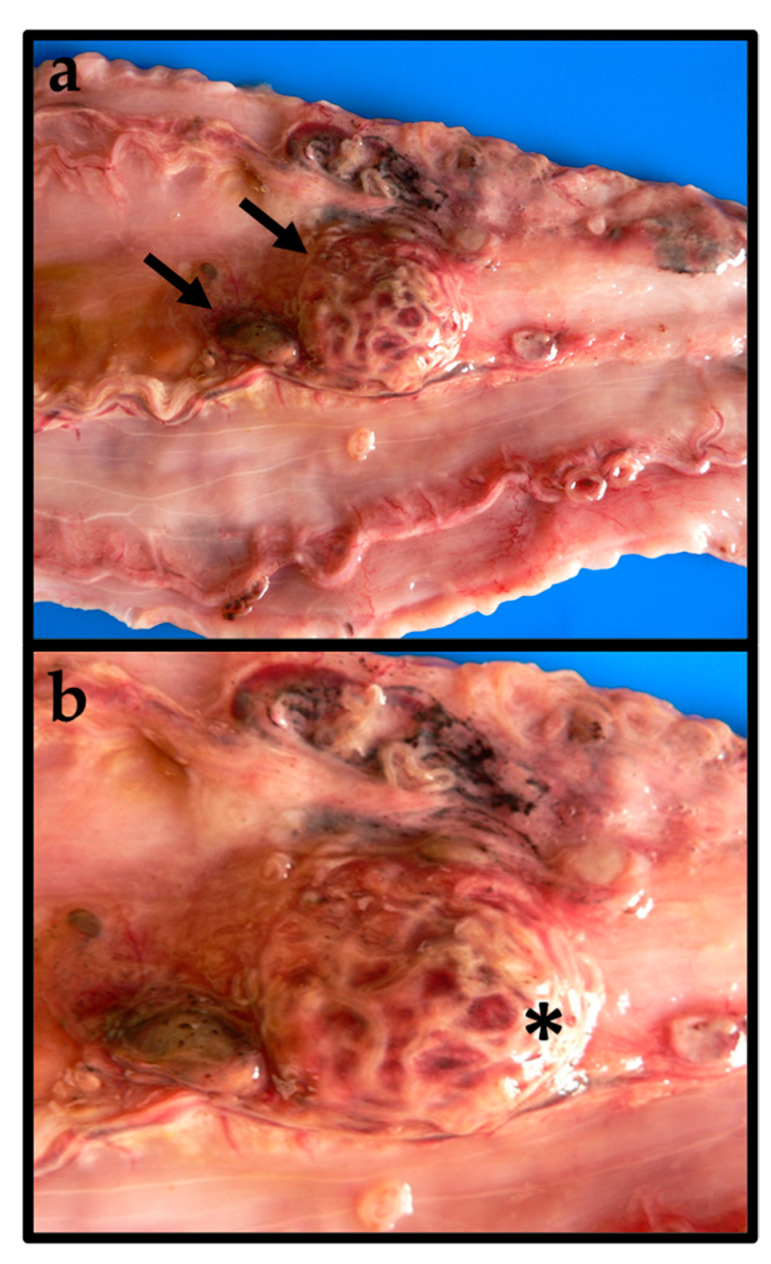
(**a**) Gastric nodular lesions in *Sphyraena viridensis* caused by *Anisakis pegreffii* (arrows). (**b**) Note the presence of numerous *A. pegreffii* larvae inside the gastric nodule (asterisk).

The larvae were found mainly attached to the serous walls of the celomic organs and within the stomach and intestine walls. In one case, a large female specimen of 4064 g, a massive larvae infection was associated with nodular lesions on the gastric wall, showing the presence of viable larvae inside (Figure 2a,b).

**Figure 3:**
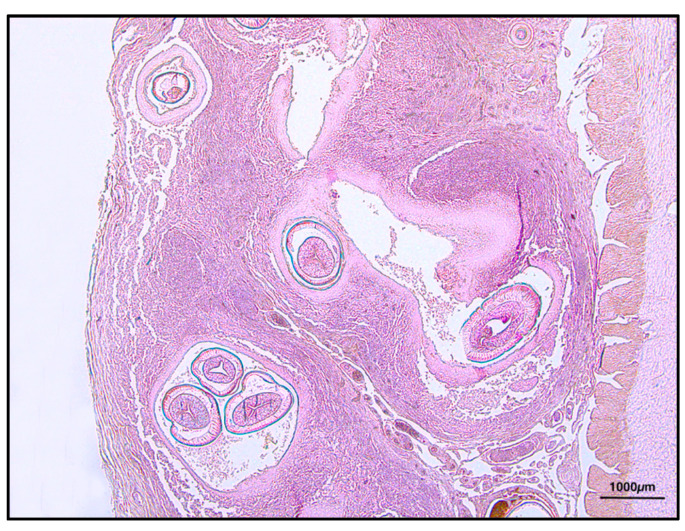
Mucosal granulomatous reaction with nematodes inside, H&E 2.5×.

By histology, several nematodes encysted through the gastric wall were found in both sub-serosa and sub-mucosa (Figure 3). Frequently, parasitic bodies were surrounded by a thick granulomatous reaction, made up of macrophages, epithelioid cells, some lymphocytes and an external connective sheet, although singular parasites appeared to be simply encysted within a thin fibrous reactive capsule.

**Figure 4:**
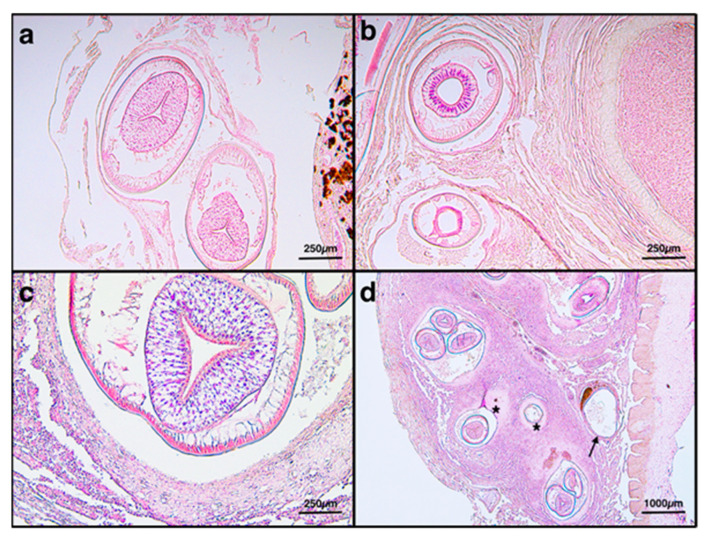
(**a**) Nematode-free larval stage found in the serosa, H&E 10×. (**b**) Encysted nematode stage, H&E 10×. (**c**) Early granuloma stage with nematodes inside a thin capsule of macrophages and epithelioid cells, H&E 10×. (**d**) Intermediate (arrow) and mature (asterisks) granuloma stages with a thick capsule surrounded by a fibrous reaction, H&E 2.5×.

**Figure 5:**
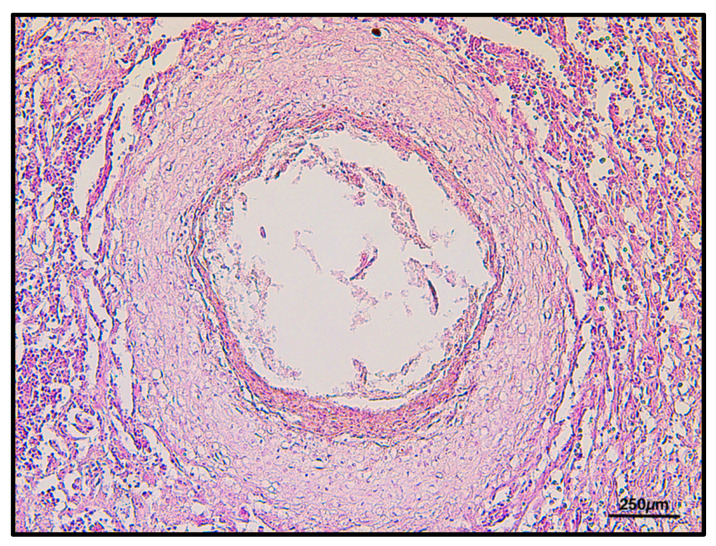
Thick granuloma reaction without parasites inside, H&E10×.

Up to five different stages of granuloma were detected (i.e., containing free larvae, encysted parasite, early, intermediate, mature (Figure 4a–d); some granulomas without parasites inside were also found (Figure 5).

### Molecular Identification of Anisakis spp.

The genomic DNA of *Anisakis* larvae collected from *S. viridensis* was successfully amplified by both primer sets identifying *18S rRNA* and *cox2* molecular markers. The obtained nucleotide sequences were submitted to GenBank database under the accession numbers OK448176 and OK483324 for the *18S rRNA* gene and *cox2* gene, respectively.

The amplification of the *18S rRNA* region produced a fragment of 926 bp; blast search showed that the *18S* DNA sequences identified in this study matched previously reported *18S rRNA* nucleotide sequences of the *Anisakis* species, *A. pegreffii* (MF072697.1 and EF180082.1), *A. simplex* (MF072711.1) and *Anisakis* sp. (U94365.1), with ∼99% identity. Unfortunately, our isolates were not assignable to species level based on *18S rRNA* sequences, since the amplified region shared the same sequence in both *Anisakis* species. A sequence of 629 pb coding for the mtDNA *cox2* gene was obtained from *Anisakis* specimens isolated in this study. It is noteworthy that the sequence analysis showed that the identified *cox2* gene matched exclusively with the *A. pegreffii cox2* sequences previously deposited in GenBank (139 hits found with E value of 0.0; 87 out of 139 showed >97% identity), therefore supporting the molecular identification of *A. pegreffii* larvae (Nematoda: Anisakidae) in *S. viridensis.* Phylogenetic analyses of the obtained *cox2* sequence with other *cox2* sequences from *Anisakis* species available in GenBank showed that our *cox2* sequence clustered together with known *cox2* sequences of *A pegreffii* previously deposited by Valentini et al. [31].

**Figure 6:**
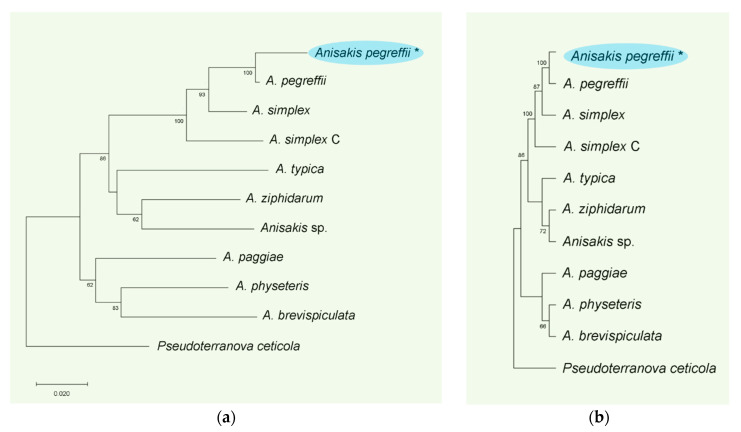
Phylogenetic relationships among *Anisakis* species as inferred by Neighbor-Joining (NJ) analysis of the *cox2* gene. The accession numbers are as follows: *A. simplex* (*s. s.*) (DQ116426), *A. pegreffii* (DQ116428), *A. simplex* C (DQ116429), *A. typica* (DQ116427), *A. ziphidarum* (DQ116430), *A. physeteris* (DQ116432), *A. brevispiculata* (DQ116433), *A. paggiae* (DQ116434) and *Anisakis* sp. (DQ116431)]. *Pseudoterranova ceticola* (DQ116435.1) was used as the outgroup to root the tree. The bootstrap values (1000 replicates) are shown at the internal nodes (>70% only); * species identified in the present study (**a**).Phylogenetic relationships among *Anisakis* species as inferred by maximum-parsimony (MP) analysis of the *cox2* gene. The accession numbers are as follows: *A. simplex* (*s. s.*) (DQ116426), *A. pegreffii* (DQ116428), *A. simplex* C (DQ116429), *A. typica* (DQ116427), *A. ziphidarum* (DQ116430), *A. physeteris* (DQ116432), *A. brevispiculata* (DQ116433), *A. paggiae* (DQ116434) and *Anisakis* sp. (DQ116431)]. *Pseudoterranova ceticola* (DQ116435.1) was used as the outgroup to root the tree. The bootstrap values (1000 replicates) are shown at the internal nodes (>70% only) (**b**).

Moreover, the phylogenetic trees from both NJ and MP analyses (Figure 6a,b) showed that the mtDNA *cox2* gene from *Anisakis* specimens of this study clustered in the same, well-supported clade (100% bootstrap value) with the *cox2* sequences of *A pegreffii* previously deposited in GenBank. The trees derived from both MP and NJ analyses showed similar topologies, with *Anasakis* species clustering in two main clades: one including *A. paggiae*, *A. physeteris* and *A. brevispiculata* and the other one including all the anisakids analyzed (*A. pegreffii, A. simplex* (*s. s*.), *A. simplex* C, *A. typica* and *A. ziphidarum*).

## 4. Discussion

The present study represents the first description of a gastric granuloma in *S. viridensis* by *A. pegreffii*. In fish, the migration of *Anisakis* larvae through the stomach wall mainly causes ulceration localized on the stomach mucosa surface, without negative effects on the physiological organ function [29]; in our case, this kind of lesion was not found. *Anisakis simplex* sensu lato is considered responsible for “Red belly syndrome” in Atlantic salmon (*Salmo salar*), characterized by hemorrhagic lesions in the area surrounding the vent, associated with the presence of non-encapsulated larvae [35], probably attributable to specific *A. simplex* tropism in *S. salar* and not superimposable on the lesion described in the present study.

In humans, ingested larvae may be expelled by digestive and peristaltic processes, but in some case, L3 larvae can penetrate the wall of the gastrointestinal tract, causing local chronic granulomatous reaction [36]. This reaction is totally macroscopically superimposable on the granulomatous lesions herein described. The immune response and adaptation described in human during this kind of infection [15,16] were not clearly described in fish, thus far, also because is not easy to perform this investigation in wild fish. From a histological point of view, human *A. pegreffii* lesions show a marked edema externally, localized in the submucosa layer often associated with abundant inflammatory infiltrate in the muscular layer, mainly composed of eosinophil granulocytes, followed by lymphocytes and plasma cells [37], whereas in *S. viridensis*, only sparse lymphocytes can be found in the muscle around granulomas. In this case, fish cannot be considered an appropriate animal model for the study of some human pathologies, as reported by Schmale et al. [38], also because the inflammatory response is different between teleosts and humans. A chronic granulomatous human lesion due to *A. pegreffii* could develop into abdominal peritonitis often associated to intestinal occlusion and abundant inflammatory reactions such as edema and fibrinous exudate [39,40]. In the present case, the gastric wall was involved, while inflammatory involvement of neighboring gastrointestinal tract, lumen occlusion and exudate reaction were not found.

The *Anisakis* larvae prevalence here observed reached much higher values (17.6 vs. 5.7%) than those reported in a previous study from other areas of the western Mediterranean Sea [9]. In agreement with the statistically significant correlation between fish body weight and parasite load found in the current study, larger fish are more likely to prey on a higher quantity and variability of intermediate or paratenic hosts. However, in some cases, as described by other authors [41], large or old fish may show a reduction in the parasitic load compared to smaller specimens, probably because they are able to develop a better immune response against new infections. The sequence and phylogenetic analyses of the mtDNA *cox2* gene supported the molecular identification of the zoonotic species *A. pegreffii*, causing gastric granuloma in *S. viridensis* for the first time. This study corroborates the value of the mitochondrial *cox2* gene as an effective molecular marker for the identification of closely related species of the *Anisakis* genus. The phylogenetic trees obtained by NJ and MP analyses showed that anisakid nematode species separate in two major clades, with *A. paggiae*, *A. physeteris* and *A. brevispiculata* grouping in a separate clade, as reported by Mattiucci et al. [40]. This study highlights that the combined morphological and molecular approaches offer a valuable tool for the identification of the *Anisakis* species.

## 5. Conclusions

In the present study, we described granulomatous lesions caused by *A. pegreffii* in *S. viridensis* from the central Mediterranean Sea for the first time, focusing on the typology of lesions and comparing them with eosinophilic granulomas described in humans.

Although at gross anatomy the lesions here observed may be superimposable to the eosinophilic granulomas described in humans, some specific histological differences of the inflammatory cells’ composition may be highlighted.

## Figures and Tables

**Table 1:** List of the primers used in this study.

Gene	Forward Primer Sequence	Reverse Primer Sequence	Size (bp)	Reference
***18S rRNA***	CGCGAATRGCTCATTACAACAGC	GGGCGGTATCTGATCGCC	926	Floyd et al. [25]
***cox2***	TTTCTAGTTATATAGATTGRTTYAT	CACCAACTCTTAAAATTATC	629	Nadler and Hudspeth [26]

## Data Availability

The data presented in this study are available on request from the corresponding author.
